# Lymphoid Progenitor Cells from Childhood Acute Lymphoblastic Leukemia Are Functionally Deficient and Express High Levels of the Transcriptional Repressor Gfi-1

**DOI:** 10.1155/2013/349067

**Published:** 2013-10-03

**Authors:** Jessica Purizaca, Adriana Contreras-Quiroz, Elisa Dorantes-Acosta, Eduardo Vadillo, Lourdes Arriaga-Pizano, Silvestre Fuentes-Figueroa, Horacio Villagomez-Barragán, Patricia Flores-Guzmán, Antonio Alvarado-Moreno, Hector Mayani, Isaura Meza, Rosaura Hernandez, Sara Huerta-Yepez, Rosana Pelayo

**Affiliations:** ^1^Oncology Research Unit, Oncology Hospital, Mexican Institute for Social Security, Avenue Cuauhtemoc 330, Colonia Doctores, 06720 Mexico City, DF, Mexico; ^2^Molecular Biomedicine Program, CINVESTAV, 07360 Mexico City, DF, Mexico; ^3^“Federico Gómez” Children's Hospital, 06720 Mexico City, DF, Mexico; ^4^Immunochemistry Research Unit, Medical Specialties Hospital, Mexican Institute for Social Security, 06720 Mexico City, DF, Mexico; ^5^UMAE “Victorio de la Fuente Narváez”, Mexican Institute for Social Security, 07760 Mexico City, DF, Mexico; ^6^“Carlos McGregor Sanchez” Hospital, Mexican Institute for Social Security, 03100 Mexico City, DF, Mexico

## Abstract

Acute lymphoblastic leukemia (ALL) is the most frequent malignancy of childhood. Substantial progress on understanding the cell hierarchy within ALL bone marrow (BM) has been recorded in the last few years, suggesting that both primitive cell fractions and committed lymphoid blasts with immature stem cell-like properties contain leukemia-initiating cells. Nevertheless, the biology of the early progenitors that initiate the lymphoid program remains elusive. The aim of the present study was to investigate the ability of lymphoid progenitors from B-cell precursor ALL BM to proliferate and undergo multilineage differentiation. By phenotype analyses, *in vitro* proliferation assays, and controlled culture systems, the lymphoid differentiation potentials were evaluated in BM primitive populations from B-cell precursor ALL pediatric patients. When compared to their normal counterparts, functional stem and progenitor cell contents were substantially reduced in ALL BM. Moreover, neither B nor NK or dendritic lymphoid-cell populations developed recurrently from highly purified ALL-lymphoid progenitors, and their proliferation and cell cycle status revealed limited proliferative capacity. Interestingly, a number of quiescence-associated transcription factors were elevated, including the transcriptional repressor Gfi-1, which was highly expressed in primitive CD34^+^ cells. Together, our findings reveal major functional defects in the primitive hematopoietic component of ALL BM. A possible contribution of high levels of Gfi-1 expression in the regulation of the stem/progenitor cell biology is suggested.

## 1. Introduction

Acute lymphoblastic leukemia (ALL), characterized by the malignant and uncontrolled proliferation of lymphoid precursor cells within bone marrow (BM), is the hematological disorder with the highest frequency in childhood and the most common cause of mortality in children worldwide [1–5]. Despite the relatively high disease control by therapeutic agents, relapses occur in approximately 20% of children due to minimal residual disease and a functional failure in the cancer surveillance mechanisms [6, 7]. Neither the precise origins of the leukemic cell, nor the biological behavior of the hematopoietic primitive cells in the leukemic setting are known. Moreover, the understanding of the mechanisms that damage the earliest steps of the lymphoid developmental program in ALL is incomplete, and the existence of specialized cancer stem cells is still a debate [8]. The identification of leukemic clones with unrelated DJ rearrangements and the presence of cytogenetic abnormalities on lineage negative cells strongly suggest the participation of primitive cells. Furthermore, data showing that only cells with immature phenotypes are capable of engraftment and leukemia reconstitution in immunodeficient mice models support this [9–12]. However, recent work has remarkably shown that diverse leukemic blast subsets with stem cell properties—the leukemia-propagating cells—can reconstitute and completely reestablish the leukemic phenotypes *in vivo *[13–15], challenging the hierarchical stem cell model for ALL [16]. In addition, the combination of genomics and xenotransplant models has indicated unsuspected genetic diversity within multiple leukemogenic subclones, consistent with the model of multiclonal evolution [17–19], outlining the importance of seeking marks from the earliest hematopoietic events in ALL and investigating the competence of stem and progenitor cells to efficiently develop and replenish the immune system of these hematological patients. 

Lymphoid cell production requires differentiation of unique primitive cells within BM [20–22]. The prospective isolation of hematopoietic stem cells (HSC) and differentiating progenitors along with the depicting of transcriptional networks and microenvironmental signals that control early cell fate decisions have been critical to the construction of developmental maps during normal hematopoiesis. It is noteworthy that the RAG1/GFP reporter mouse has been extremely useful to define the earliest progenitors (ELP) that initiate the lymphoid differentiation program [23–25]. In BM, the ELP fraction is responsible for production of key components of the innate immune system, including plasmacytoid dendritic cells (pDC) and interferon producing killer dendritic cells (IKDC) [24–26]. They also give rise to more differentiated common lymphoid progenitors (CLP), which generate precursors of B lymphocytes and NK cells while losing the possibility of differentiating into the rest of the lineages. Human hematopoiesis is generally consistent with the process in mice with some differences in cell phenotypes. Normal development of myeloid and lymphoid human cells from CD34^+^ HSC involves a stepwise progression of differentiating cells [27, 28]. Myeloid precursor cells are produced by multipotent progenitor cells (MPP); whereas, the earliest lymphoid progenitors might be directly derived from HSC and have been recently designated as multilymphoid progenitors (MLP). A counterpart of CLP efficiently differentiates into B and NK cells [29]. Downstream, committed precursors sequentially originate early B-, proB-, preB-, and immature B-cells, until the production of mature B-cells that are eventually exported to the peripheral lymphoid tissues. Primitive BM cells can assume alternative fates in response to genetic abnormalities or environmental cues [2, 30–32], suggesting that the relative stability of a lineage could undergo adjustments in the setting of infection and disease. 

In order to investigate the functional properties of stem and progenitor cells in B-cell precursor ALL, we have examined their ability to proliferate and undergo multilineage differentiation, showing a defective *in vitro* cell development from early BM lymphoid progenitors. To inquire into molecular mechanisms that may be involved in the poor proliferative and differentiation potentials of ALL-lymphoid progenitors, the transcription of some genes relevant to the quiescence status of hematopoietic primitive cells was evaluated. Of note, transcriptional repressor growth factor independence-1 (Gfi-1), formerly identified as a bifunctional regulator of hematopoietic differentiation [33–35], was highly displayed by ALL progenitor cells. Its nuclear distribution suggests a possible biological role in the pathogenesis of this disease.

## 2. Materials and Methods

### 2.1. Patient Characteristics and Sample Collection

 Thirty children referred to the “Federico Gomez” Children's Hospital (Mexico City, Mexico) and diagnosed with B-cell precursor acute lymphoblastic leukemia were included in the study. Among them, 24 patients fulfilled the criteria, by blood cell count, for high-risk disease, whereas 6 for standard-risk. Within the high-risk group, 58.3% of the patients were female and 41.6 were male, while the standard risk group included 66.6% female and 33.3% male patients. The median age values were 7.1 yr (3 mo–14.9 yr) and 5.3 yr (2.1 yr–8 yr) for the high-risk and standard-risk group, respectively. BM specimens were collected by aspiration before any treatment, respecting international and institutional guidelines. All procedures were approved by the Ethics, Research and Biosafety Committee of the “Federico Gómez” Children's Hospital (Registry HIM/2009/033) and by the National Committee of Scientific Research at the Mexican Institute for Social Security (Registry R-2010-785-012). Control BM specimens were obtained from healthy children undergoing minor orthopedic surgery. All samples were collected after informed consent from the parents.

### 2.2. Isolation of Precursor Cells

 Mononuclear cells (MNC) from B-cell precursor ALL patients were prepared by Ficoll-Paque Plus (GE Healthcare Bioscience) gradient separation. CD34^+^ fraction containing HSC and progenitor cells was enriched from MNC using the human CD34 progenitor cell isolation kit (Miltenyi Biotec) according to the manufacturer's instructions. Cells were counted before analyses, and the purity of the cell fractions was confirmed by flow cytometry. Cell samples from all studied control and ALL patients were individually manipulated and analyzed. In order to identify biological heterogeneity on the basis of the primitive cell behavior, and to limit heterogeneity due to patient diversity and/or disorder variations, no sample pooling was performed for any of the experimental strategies. 

### 2.3. Hematopoietic Colony Assays

 Hematopoietic progenitor cells capable of forming colonies *in vitro* (colony-forming cells, CFC) were assayed in methylcellulose-based semisolid cultures (MethoCult, STI), as described [36, 37]. 

### 2.4. Long Term Culture-Initiating Cells (LTC-IC)

 LTC-IC numbers were determined from CFC assays of long term cultures using the M2-10B4 stromal cell line as feeder layer. Cultures were sustained for 5 weeks, cells harvested, and proportions plated in semi-solid cultures for quantification of CFC [37].

### 2.5. Flow Cytometry

 Identification of stem and progenitor cell fractions from MNC was first based on their expression of the enzyme aldehyde dehydrogenase (ALDH) according to the manufacturer's instructions (Aldefluor, Stem Cell Technologies). 

 Phenotyping of primitive cells was performed by five-color flow cytometry on a FACSAria flow cytometer (BD Biosciences) or on a CyAn flow cytometer (Beckman Coulter), after staining with anti-lineage markers (CD3, CD8, TCR, CD56, CD14, CD11b, CD20, CD19, and CD235a), anti-CD34, anti-CD45RA, anti-CD10, and anti-CD7 fluorochrome-conjugated antibodies (BD Pharmingen). Hematopoietic stem cells were identified as Lin^−^CD34^+^CD45RA^−^, while the fraction containing multipotent and multilymphoid progenitors (MPP and MLP, resp.) was identified as Lin^−^CD34^+^CD45RA^+^. MPP were further subfractionated into T-cell progenitors (TP, Lin^−^CD34^+^CD45RA^+^CD7^+^CD10^−^), early lymphoid progenitors (ELP, Lin^−^CD34^+^CD45RA^+^CD7^+^CD10^+^), and common lymphoid progenitors (CLP, Lin^−^CD34^+^CD45RA^+^CD7^−^CD10^+^). For cell co-cultures, the fraction containing MPP and MLP was highly purified by multicolor flow cytometry/sorting using a FACSAria flow cytometer (BD Biosciences). Cell purity was confirmed by postsorting analysis. After lymphoid co-cultures, differentiated cells were counted to calculate cell frequencies and yields per input progenitor prior to staining with directly conjugated fluorescent antibodies (Invitrogen and BD Pharmingen). Newly produced B-cells were identified as CD3^−^CD19^+^, while NK cells as CD3^−^CD19^−^CD56^+^. Within the fraction CD3^−^CD19^−^CD56^−^, cells expressing CD11c were considered conventional dendritic cells, whereas the BDCA2 expressing cells were classified as plasmacytoid dendritic cells. Analysis of flow cytometry data was performed using the FlowJo 7.6.1 software.

### 2.6. Cell Cycle and Proliferation Analyses

 7-aminoactinomycin (7-AAD) was used to quantitatively assess DNA content as it binds stoichiometrically to DNA. After membrane staining with an anti-CD34 monoclonal antibody (mAb), control and ALL cells were permeabilized and fixed prior to 7-AAD staining, according to an established protocol (BD Biosciences). CD34^+/−^ cells were classified into phases of cell cycle by using the Cell Cycle Flow Cytometry Analysis Software (FlowJo, Tree Star, Inc.), which fits the DNA histogram into mathematical distributions through algorithms and automatically calculates the cell frequencies in G_1_/G_0_, S, and G_2_/M (http://www.flowjo.com/v9/html/cellcycle.html).

For the bromo-2′-deoxyuridine (BrdU) incorporation assay, Lin^−^CD34^+^ cells were cultured for 120 hours with BrdU to a final concentration of 10 *μ*M, in supplemented *α*-MEM containing 10% FCS. Cells were intracellularly stained with a PE-labeled mAb anti-BrdU to visualize BrdU in the studied primitive cells (BrdU flow kit, BD Biosciences) and analyzed on a FACSCalibur flow cytometer (BD Biosciences).

### 2.7. TUNEL Assay

 To detect DNA fragmentation as a result of apoptosis in ALL primitive cells, the terminal deoxynucleotidyl transferase dUTP nick end labeling (TUNEL) assay was performed (*In situ* cell death detection kit, POD Roche). Briefly, cell smears from sorted HSC, ELP, and CLP were incubated for 1 hour with terminal transferase enzyme (TdT) 1 : 50 in a buffer containing fluorescein-conjugated oligonucleotides. Upon several washes, antifluorescein antibody was added and the reaction developed with diaminobencidine reagent (Dako). Cells were counterstained with hematoxylin and dehydrated for further coverslipping procedures.

### 2.8. Stromal Cell Cocultures

 To evaluate the ability of ALL progenitors to undergo multilineage differentiation, sorted cells were co-cultured on MS-5 stromal cell monolayers for 30 days in lymphoid conditions. The *α*-modified essential medium (*α*-MEM) was supplemented with 10% fetal bovine serum, 1 ng/mL Flt3-L (FL), 2 ng/mL SCF, 5 ng/mL IL-7, and 10 ng/mL IL-15 (Peprotech) and contained 100 U/mL penicillin plus 100 mg/mL streptomycin. Co-culture plates were incubated at 37°C in a humidified atmosphere of 5% CO_2_. This controlled system promotes the efficient differentiation of hematopoietic stem/progenitor cells towards B-cells, NK cells, and lymphoid-related dendritic cells. Control cells were cultured in the same experiment for comparison. Absolute numbers of differentiated cells per lymphoid progenitor were analyzed and presented as yields per input progenitor.

### 2.9. Real-Time PCR Analysis of Gene Expression

 To determine the expression level of a number of genes known to be involved in regulation of the quiescence status of normal early hematopoietic progenitor cells, total mRNAs from Lin^−^CD34^+^ ALL cells were isolated by Trizol (Invitrogen) and cDNAs were prepared using Moloney murine leukemia virus reverse transcriptase (Invitrogen). Quantitative RT-PCR was performed using a LightCycler 2.0 capillary instrument (Roche). According to the Design Center from Roche: http://www.roche-applied-science.com/webapp/wcs/stores/servlet/CategoryDisplay?catalogId=10001&tab=Assay±Design±Center&identifier=Universal±Probe±Library&langId=-1, the specific forward and reverse oligonucleotide primers were Bcl-2 (5′-CCCCTATCAAGCAGGAATACTCTA-3′; 3′-GCCGTTCCTCGTTTTATCCT-5′), Bmi-1 (5′-GCATCACAGTCATTGCTGCT-3′; 3′-TTCTTTGACCAGAACAGATTGG-5′), c-Myc (5′-CGAAGCAGCTCTATTTCTGGA-3′; 3′-GAACCAGAGAAACCTAACAGTGC-5′), FOXO3A (5′-TCTCCAGTTCCCTTGGGTATTA-3′; 3′-ACCTTCCCATTATGCACTGG-5′), Gfi-1 (5′-CGTGAGGGGTGGAGAAGAC-3′; 3′-CCTCTTGTGCCCAGCACT-5′), HOXB4 (5′-AGCGGTTGTAGTGAAATTCCTT-3′; 3′-CTGGATGCGCAAAGTTCAC-5′), p19 (5′-CCCCTCTCTTCTGATACATAACCA-3′; 3′-CTCAGGACCTCGTGGACATC-5′), p21 (5′-TGGGTCTGCTCTAAGTCAAGC-3′; 3′-ACAAGCACACAGAGCTAGTAGTGG-5′), p53 (5′-AACATCTCGAAGCGCTCAC-3′; 3′-CCCCAGCCAAAGAAGAAAC-5′), PTEN (5′-TCCAGATGATTCTTTAACAGGTAGC-3′; 3′-GGGGAAGTAAGGACCAGAGAC-5′), *β*-actin (5′-CCAGAGGCGTACAGGGATAG-3′; 3′-CCAACCGCGAGAAGATGA-5′). The relative gene expression was calculated using *β*-actin cDNA as an endogenous control.

### 2.10. Western Blotting

 Total protein extracts from ALL CD34^+^ cells were obtained by using lysis buffer and a protease inhibitor cocktail (Roche), while nuclear protein extracts were obtained with lysis buffer plus a manual device for nuclear extraction. Proteins were separated on 12% SDS-PAGE gels and transferred to nitrocellulose membranes, where the incubation with anti-Gfi-1 rabbit antibody took place overnight. HRP-conjugated secondary anti-rabbit IgG was used before visualization with the enhanced chemiluminescence kit (GE Healthcare) and documentation in a ChemiDoc system (BioRad). Blots were striped using a buffer containing 2% SDS, 100 mM 2-mercaptoethanol, and 50 mM Tris pH 6.8 and reincubated with *α*-actin (43 kDa) as housekeeping control protein, or LaminB1 (67 kDa) as nuclear protein marker, followed by the secondary antibody.

### 2.11. Immunocytochemistry

 ALL and control CD34^+/−^ cells were fixed with 4% paraformaldehyde and hydrated with PBS. After endogenous peroxidase blocking, cells were incubated with 2% normal swine serum (Vector) for 2 hours at room temperature and incubated overnight with anti-Gfi-1 antibody (Abcam) or with normal rabbit IgG (Santa Cruz Biotechnology) as an isotype control. LSAB + System-HRP (Dako) was used instead of a secondary antibody. The reaction was developed with diaminobencidine (Dako) and upon washing, counterstained with hematoxylin and dehydrated for coverslipping. 

### 2.12. Immunofluorescence Microscopy

ALL and control CD34^+^ cells were fixed using 4% paraformaldehyde and hydrated with PBS. Permeabilization was performed with 0.01% Triton X-100 (Bio-Rad) and blocking with 2% BSA for 30 minutes at 37°C. Anti-Gfi-1 antibody (Abcam) was incubated for 1 hour at 37°C followed by incubation with goat anti-rabbit Alexa 488 (Invitrogen) antibody. Slides were mounted with vectashield with propidium iodide (Vector). Overlay of images from the different fluorescent channels was performed using ImageJ software (NHI) and Image Pro Plus software.

### 2.13. Statistics

The Prism software, version 5.01 (GraphPad) was used for statistical analysis. Comparisons between groups were performed with the unpaired *t-*test. *P* values were 2-tailed and considered significant if less than 0.05.

## 3. Results and Discussion

### 3.1. The Functional Activity of the Most Primitive Cell Populations from ALL Bone Marrow Is Dramatically Impaired *In Vitro*

We are interested in the biology of early human progenitors that first initiate the lymphoid program in B-cell precursor ALL pediatric patients at the presentation of the disease. In order to investigate the functional abilities of these rare cells, flow cytometry phenotype analyses, bromo-deoxyuridine incorporation assays, and stromal cell cocultures were conducted. Our findings strongly suggest *in vitro* defective proliferation and differentiation potentials of ALL lymphoid-early and committed progenitors, which might result from their intrinsic properties or special environmental requirements.

We have conducted this study with 30 ALL cases, with 24 of them classified as high risk on the basis of their leukocyte numbers, and only 1 with apparent genetic abnormalities. Mononuclear cells were taken from BM, and CD10-CD19-CD34 triple stainings were performed to confirm the diagnosis. While control BM from nonhematological patients had less than 5% CD34^+^CD19^+^CD10^+^ cells, near to 70% was seen in most ALL BM. All of the studied samples were classified as Pro- or early Pre-B-ALL (data not shown). 

**Figure 1:**
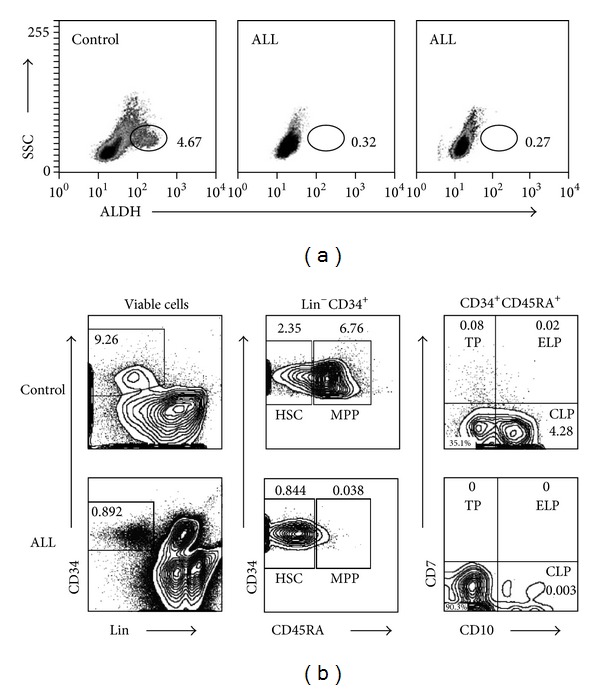
Hematopoietic and progenitor cell frequencies are critically reduced in acute lymphoblastic leukemia (ALL). The primitive cell fraction was identified in control and ALL bone marrow (BM) on the basis of its functional expression of aldehyde dehydrogenase. Two ALL samples and one control are shown (a). Mononuclear cells from ALL BM were resolved in stem and lymphoid progenitor populations according to CD34, CD45RA, CD7, and CD10 expression, and their frequencies were calculated and compared to normal counterparts (b). HSC were identified as Lin^−^CD34^+^CD45RA^−^ and MPP as Lin^−^CD34^+^CD45RA^+^. MPP subfractionation gave rise to TP (Lin^−^CD34^+^CD45RA^+^CD7^+^CD10^−^), ELP (Lin^−^CD34^+^CD45RA^+^CD7^+^CD10^+^), and CLP (Lin^−^CD34^+^CD45RA^+^CD7^−^CD10^+^). HSC: hematopoietic stem cells; MPP: multipotent and multilymphoid progenitor cell fraction; TP: T-cell progenitors; ELP: early lymphoid progenitors; CLP: common lymphoid progenitors.

Based on their aldehyde dehydrogenase enzymatic activity, the stem/progenitor cell pool consistently showed a substantial reduction −10-fold or more in cell frequencies from B-ALL (Figure 1(a)). Accordingly, BM fractioning into stem cells and the various multipotent progenitors indicated a significant contraction in the whole compartment, including early lymphoid progenitor, T-cell progenitor, and common lymphoid progenitor populations (Figure 1(b)). Interestingly, among childhood hematological neoplastic disorders, acute myeloid leukemia has also been shown to have critical variations in frequencies of BM CD34^+^ primitive cells [2].

To evaluate the stem cell content and activity in B-ALL, we performed limiting dilution assays in long term cultures and were able to quantify clonal long term culture-initiating cells (LTC-IC) after 10 wk. Although the number of primitive cells from the studied ALL patients was critically reduced as previously shown by cytometric strategies, we did not pool cell samples for any of the cellular differentiation and proliferation assays. Of note, these rare primitive precursors were capable of differentiating, but their blood cell production potentials was decreased by half (Table 1). Colony-forming cell assays are helpful to assess functional activity of the downstream developmental stage, and under our particular myeloid-culture conditions, the myeloid potential was examined. In contrast to the modest reduction of LTC-IC content, lower numbers of colony-forming units were apparently compared to their normal counterparts, indicating major deficiencies in the multipotent/oligopotent compartment rather than in the stem cell population (Table 1). These observations are in keeping with previous reports from Martínez-Jaramillo and colleagues on adult lymphoblastic leukemia, showing that equivalent cell populations possess severe deficiencies in proliferation and expansion [38]. Moreover, a defective *in vitro* growth has also been demonstrated in both pediatric and adult myeloid leukemia types [39–41].

**Figure 2:**
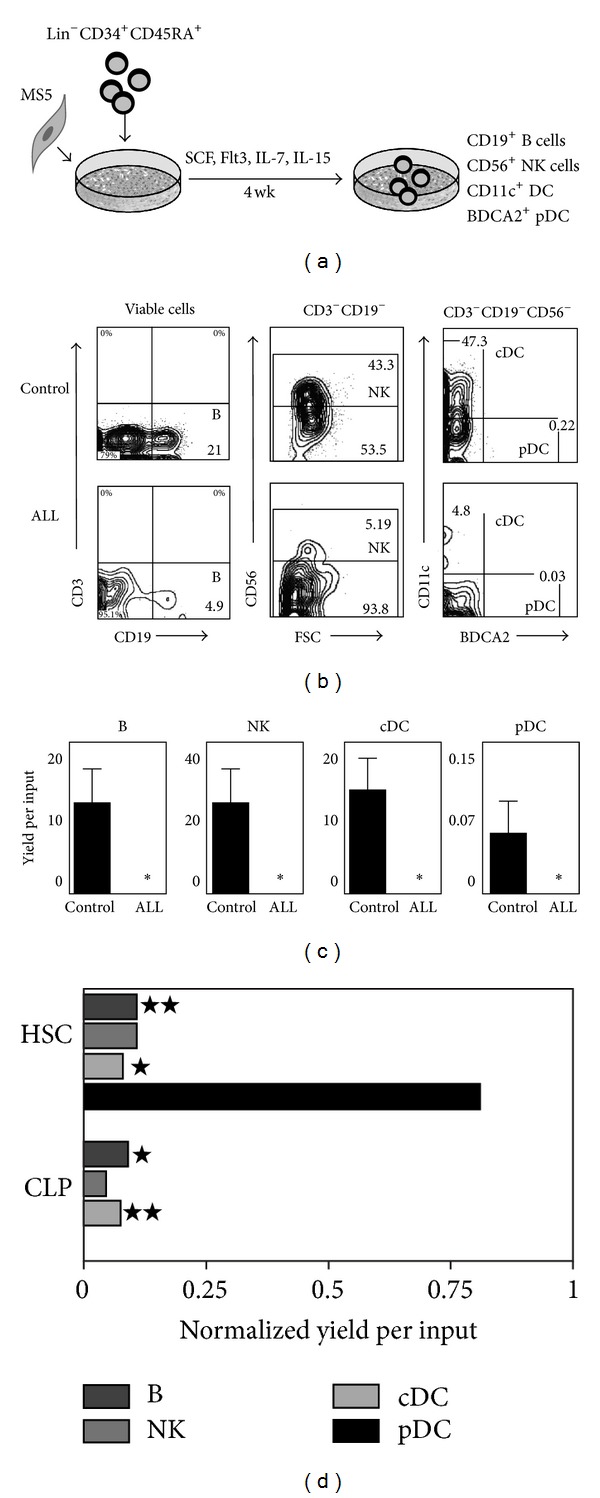
Defective lymphoid-differentiation potential of primitive bone marrow cells in ALL. Progenitor cell fractions were isolated from control and ALL bone marrow (BM) and placed in lymphoid cocultures for 4 wk on MS-5 stromal cells with SCF, FL, IL-7, and IL-15 before flow cytometry analyses. The general culture approach is shown (a). Cell frequencies of recovered B, NK, conventional dendritic, and plasmacytoid dendritic cells from Lin^−^CD34^+^CD45RA^+^ differentiation cultures were further calculated, and their absolute numbers tabulated as yield per input progenitor ((b) and (c)). Highly purified HSC and CLP cells were cultured in the same conditions and the resulting yields were normalized to control values (d). HSC: hematopoietic stem cells; CLP: common lymphoid progenitor.

No recapitulation of leukemia was exhibited by any of the conducted culture conditions. Furthermore, the 4-week lymphoid co-culture results (Figure 2(a)) strongly suggested that B-ALL Lin^−^CD34^+^CD45RA^+^ progenitors can differentiate toward B-, NK-, conventional dendritic- (cDC), and plasmacytoid dendritic- (pDC) lymphoid cells. However, the normal cell differentiation potential is critically compromised in leukemia, as shown by the scant cell frequencies and yield per input values (Figures 2(b) and 2(c)). The same was true when we investigated the cell production abilities of highly purified hematopoietic stem cells (HSC) or common lymphoid progenitors (CLP) for most lymphoid maturing cells (Figure 2(d)), suggesting that a strong negative regulation may occur throughout the pathway, from the earliest lymphoid-primed populations to the committed CLP.

Undoubtedly, an extension of these studies to additional functional assays (including xenotransplantation) and gene expression analyses will aim to get a better picture of the presumably “inactive” early lymphoid compartment in ALL BM.

The cell cycle status and apoptosis rates of primitive cells may provide relevant information about their cell production efficiency. In our current hierarchical model of normal hematopoietic development, HSC and early lymphoid progenitors are mostly quiescent and divide intermittently, whereas more committed cells might reside far from the quiescent niche and have distinct requirements and proliferation rates [25, 42, 43]. In steady state, none of these populations is homogeneous, and at any given time they exist in two kinetic states that are tightly controlled. 

**Figure 3:**
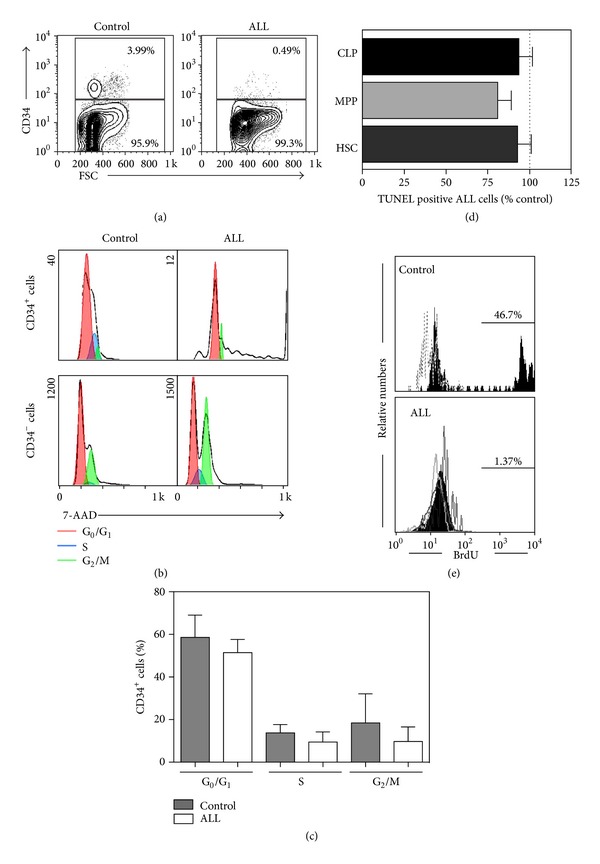
Normal apoptosis rates but poor proliferative capabilities mark the early steps of lymphoid development in ALL. Mononuclear cells from control and ALL bone marrow (BM) were fractionated according to CD34 expression and analyzed by flow cytometry (a). The cell cycle status of primitive CD34^+^ cells and of CD34^−^ maturing cells (as indicated from the flow cytometry gates) were examined by 7-AAD staining (b). The CD34^+^ cell frequencies in G_0_/G_1_, S, and G_2_/M phases of the cell cycle were tabulated (c). Apoptosis rates in highly purified HSC, MPP, and CLP populations were assessed by TUNEL (d), while the proliferative disadvantage in the total fraction of progenitor cells in ALL BM was highlighted by means of BrdU incorporation in stromal cell-free conditions (e). HSC: hematopoietic stem cells; MPP: multipotent progenitor; CLP: common lymphoid progenitor.

Here, cell cycle distribution of ALL cells was investigated by flow cytometry in the pool of CD34^+^ multipotent BM cells, including stem and progenitor cell populations. Intracellular 7-AAD staining profiles indicated that the very scant fraction of CD34^+^ cells is to some degree heterogeneous with respect to cell cycle phases, with no obvious discrepancies between their G_0_/G_1_ cell frequencies and the control counterparts (Figures 3(a)–3(c)). Slight decreases in the proportions of CD34^+^ cells in S and G_2_/M phases of the cell cycle were recorded in ALL BM, concomitant with higher cell death in some samples that resulted of no statistical significance.

Furthermore, the cyclin-dependent kinase inhibitors (cdki) p21 and p27, which has been shown to play a role in maintaining stem cells in quiescence and self renewing state, did not exhibit a difference by flow cytometry (data not shown). 

In contrast, the DNA content analyses of CD34^−^ committed precursors exhibited lower frequencies within the G_0_/G_1_ phase and increasing proportions in the S and G_2_/M phases, suggesting that they were more actively coursing through cell cycle or arrested in the G_2_ phase (Figure 3(b), lower panels). Evaluation of the mitotic index or the status of the Chk1-Cdc25C-Cdk1 G_2_ checkpoint signaling pathway in this maturing cell population may help to further distinguish arrested G_2_ cells from mitotic cells.

However, when including all studied patients, CD34^−^ cell frequencies in G_2_/M were 2–34% in ALL and 3–28% in control BM (not shown), indicating a normal behavior of developing cells from ALL individuals with no apparent chromosome aberrations. Cell lysis was not evident as cells with a sub-G_1_ DNA content were less than 5%. 

The possibility of an increased rate of apoptosis as an explanation for the limited CD34^+^ cell abundance in ALL was explored by a series of TUNEL experiments with no perceptible disparities when compared to their normal counterparts (Figure 3(d)). On the contrary, the proliferative capability marked an extreme disadvantage from B-ALL primitive cells (Figure 3(e)). While near to 50% of the normal source cells have incorporated BrdU in 120 h, no proliferation at any rate was documented in ALL patients (Figure 3(e)). 

Together, these data suggest that lymphoid progenitor cells from childhood acute lymphoblastic leukemia are functionally deficient, and that the observed relative inactivity might be independent of further expansion of committed ALL-precursor cells. 

### 3.2. High Expression Levels of the Transcriptional Repressor Gfi-1 Are Detected in Functionally Deficient Lymphoid Progenitor Cells from ALL Individuals

**Figure 4:**
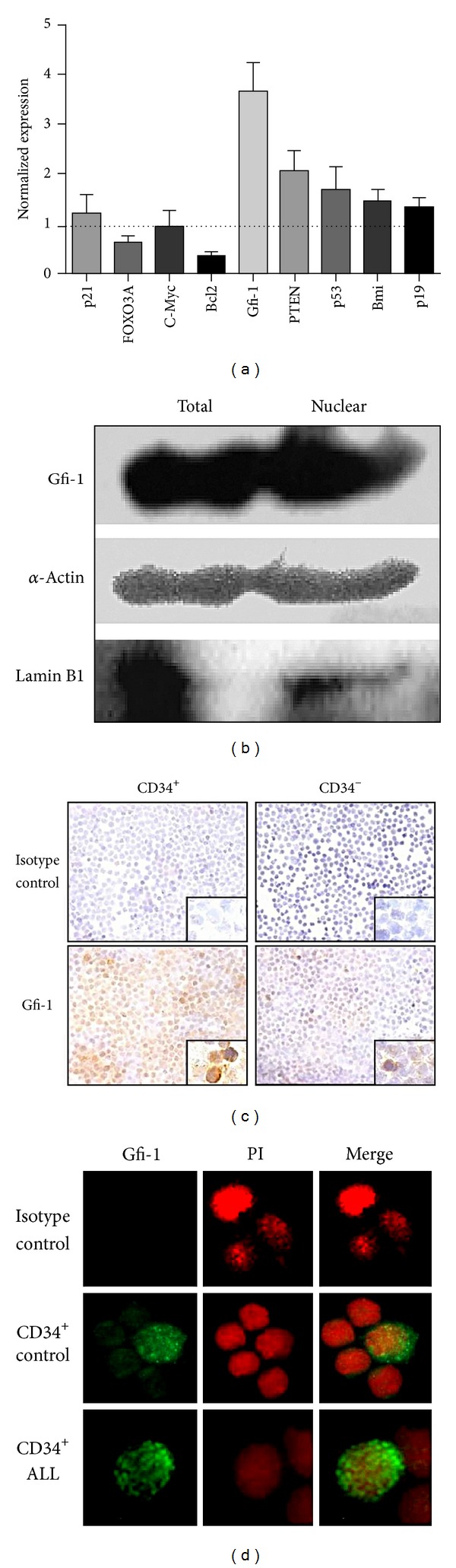
The transcriptional repressor growth factor independence-1 (Gfi-1) is highly expressed in progenitor cells from leukemic individuals. A number of transcription factors and cell cycle genes, including p21, FOXO3A, c-Myc, Bcl2, Gfi-1, PTEN, p53, Bmi, and p19, were investigated by real time RT-PCR in Lin^−^CD34^+^ cells from ALL BM, and their expression values normalized to control cells (a). The protein production of the transcriptional repressor Gfi-1 (45 kDa) was demonstrated in both nuclear and total extracts from ALL CD34^+^ cells by Western blotting. As loading control protein and as nuclear protein marker, *α*-actin (43 kDa) and LaminB1 (67 kDa) were included, respectively (b). Immunocytochemistry approach showed the highest protein expression of Gfi-1 in precursor CD34^+^ cells (c), where the nuclear location was confirmed by immunofluorescence microscopy (d).

Hematopoietic regulation of early cell fate decisions by transcriptional activities of regulatory factors that control cell quiescence and self-renewal has been extensively investigated in the last few years. The combinatorial action of specific proteins, including cell cycle inhibitors, transcription factors, antiapoptotic molecules, and oncogene products, determine both the maintenance of the stem cell pool and the ultimate commitment of hematopoietic progenitor cells [42]. The growth, differentiation, and survival of HSC have been shown to depend on the relative basal expression level of cyclins, cyclin-dependent kinases (cdks), and cdk-inhibitors (cdkis) [25, 44]. HSC cyclins are negatively regulated by cyclin-dependent kinases inhibitors (cdki). Of special interest has been the cdki p21, which is highly expressed in the quiescent HSC-like fraction of BM cells and restricts HSC entry into cell cycle, thereby imposing limits on their pool size and preventing their exhaustion [45, 46]. Many transcription factors such as c-Myb, GATA-2, Gfi-1, FOXO3A, PTEN, Bmi-1, and those of the homeobox (Hox) family have been shown to be additional key players in the proliferation and differentiation of early BM progenitor cells [47–53]. We now show that among these transcription factors with repression and activation roles in hematopoiesis, the transcriptional repressor growth factor independence-1 (Gfi-1) was significantly upregulated by Lin^−^CD34^+^ cells from B-ALL patients compared to control individuals, as shown by real time RT-PCR analyses (Figure 4(a)). Gfi-1 can regulate gene promoter activities through genetic and epigenetic mechanisms and has attracted the hematologist attention because its pivotal role in hematopoietic lineage decisions through the negative regulation of proliferation and differentiation of maturing cells [33, 34, 54, 55]. Moreover, its survival-inhibitory effect in myeloid leukemia cells has opened an exciting area that may impact therapeutic novel designs [35, 56]. Our analyses provide important information about the cell compartment where Gfi-1 is most expressed in ALL BM, showing a preferential display by primitive CD34^+^ cells over differentiating precursors (Figure 4(c)). Of relevance, Western blotting and fluorescence microscopy analyses showed an active transcriptional status of Gfi-1, as it was clearly detected inside nuclei (Figures 4(b) and 4(d)). 

Gfi-1 might be working in a dual fashion in BM cells from ALL: repressing early progenitors while activating maturing B-cell precursors. Further investigations using silencing or blocking strategies may help to prove a cause/effect relationship and to implicate the transcriptional repression by Gfi-1 in the regulation of human HSC and progenitor cell fates in leukemia. In addition, addressing Gfi-1 binding to a number of gene promoters that are relevant to B-cell production and/or maintenance is fundamental to understand the mechanisms involved in proliferation and differentiation of progenitor cells in ALL. Descriptive *in silico *analyses strongly suggest a number of putative targets for Gfi-1, including PU.1, IKAROS, RAG-1, PAX5, WNT3A, WNT5A, and STAT5, among others (data not shown; TESS: http://www.cbil.upenn.edu/tess). On the other hand, although a possible role of the leukemic microenvironment in the triggering of lineage instability in ALL is still unclear, the observed slow activity of early progenitors might be related to extrinsic factors via overfunctional transcriptional repressor molecules [57]. The actual mechanisms responsible for the *in vitro* defective behavior of B-cell progenitors remain to be elucidated and will certainly contribute to our knowledge about biology of acute lymphoblastic leukemia. 

## 4. Concluding Remarks

The mechanisms of damage to the program of lymphoid development, and the intercommunication of primitive cells with microenvironmental cues that favor the growth of leukemia cells in ALL, are still unclear. Strikingly, when the identity and function of the most primitive lymphoid cell populations from B-cell precursor ALL bone marrow were examined in controlled culture systems, a substantial deficit in their *in vitro* differentiation and proliferation potentials was recorded, suggesting a growth dependency on the leukemic milieu and a relative loss of functions of the hematopoietic and immune systems from their origins. Intrinsic mechanisms that may contribute to the inferior cell output include those provided by factors with a regulatory role in maintenance and cell cycle entry of hematopoietic stem and progenitor cells. Accordingly, high expression levels of the transcriptional repressor Gfi-1 in early cell fractions may have implications in the extent of normal differentiation through regulation of lymphoid related genes and microenvironmental factors. The presumptive implication of Gfi-1 in the regulation of human HSC and progenitor cell fates will be supported by future investigations using silencing strategies. Knowledge of early lymphoid development in ALL setting provides new insights about the immune system whose importance is critical during chemotherapy, infections, and transplantation procedures in these patients.

## Figures and Tables

**Table 1:** Reduced stem and progenitor cell content in ALL bone marrow.

	Control		ALL	
	LTC-IC	CFC	LTC-IC	CFC
MNC	0.01073 (0.0098–0.0266) 1 LTC-IC/7760 ± 2020	0.0740 (0.064–0.096) 1 CFC/1331 ± 116	0.005425 (0.00475–0.01125) 1 LTC-IC/16124 ± 3696	0.0010** (0.0–0.010) 1 CFC/20494 ± 4165

CD34^+^		1.20 (1.16–1.54) 1 CFC/78 ± 6		0.0** (0.0–0.29) 1 CFC/422 ± 77

Limiting dilution assays were conducted to determine the number of long term culture initiating cells (LTC-IC) and colony-forming cells (CFC) within mononuclear and CD34^+^ cell fractions from bone marrow (BM) acute lymphoblastic leukemia (ALL).

The results represent median (range) values and correspond to the percentage of LTC-IC and CFC within mononuclear or CD34^+^ cell fractions.

**Significantly lower (*P* < 0.01) than in control BM.
